# Functional epidermal growth factor gene polymorphisms and risk of gastric cancer

**DOI:** 10.3892/ol.2012.1041

**Published:** 2012-11-22

**Authors:** ZHEN ZHAN, YAJUN CHEN, JUAN WU, JUNFENG ZHANG, SHUJUAN TONG, CHUNBING ZHANG, YAPING YANG

**Affiliations:** 1 Department of Chinese and Western Integrative Medicine, Nanjing University of Chinese Medicine, Nanjing 210046;; 2 Department of Clinical Laboratory, First Affiliated Hospital of Nanjing University of Chinese Medicine, Nanjing 210029, P.R. China

**Keywords:** epidermal growth factor polymorphisms, gastric cancer, Chinese population, molecular epidemiology

## Abstract

Epidermal growth factor (EGF) is critical in cancer progression and various genotypes of the EGF gene have been reported to be correlated with susceptibility to gastric cancer; however, the results are unclear. The aim of this study was to explore the associations of functional EGF gene polymorphisms and risk of gastric cancer in a Chinese population. We genotyped seven functional single-nucleotide polymorphisms (SNPs) of the EGF gene [rs3756261, rs11568835 and rs4444903 in the promoter region; rs11568943, rs2237051 and rs11569017 in the non-synonymous exon region and rs3733625 in the 3′ untranslated region (UTR)] in a hospital-based case-control study, including 387 gastric cancer cases and 392 healthy controls by polymerase chain reaction-ligation detection reaction (PCR-LDR) methods. We found that individuals carrying the AG/GG genotype of rs2237051 (Ile to Met) in the exon region had an increased risk of gastric cancer (adjusted OR=1.577; 95% CI=1.163–2.138), compared with the wild-type homozygous AA genotype. The heterozygous AG/GG genotype of rs3733625 in the 3′UTR demonstrated a protective effect (adjusted OR=0.690; 95% CI=0.501–0.951), compared with the homozygous AA genotype. In addition, the effects of the two SNPs were more evident in intestinal gastric cancer (P<0.05); however, this was not case for the diffuse type (P>0.05). These findings indicate that a change in amino acids from isoleucine to methionine of rs2237051 and rs3733625 in the 3′UTR may contribute to the etiology of intestinal gastric cancer in the Chinese population.

## Introduction

Gastric cancer remains the world’s second most common malignancy, accounting for a large proportion of cancer cases in Asia, Latin America and certain countries in Europe (1). However, the etiology of gastric cancer is not well-understood. Epidemiological studies have suggested that environmental exposures, including a salty diet, tobacco smoking and *Helicobacter pylori* infection, have an effect on the development of gastric cancer (2,3). In recent years, accumulating evidence suggests that genetic alternation may play an important role in gastric carcinogenesis.

Epidermal growth factor (EGF), first isolated in 1962, has a number of biological functions. When binding with EGF-receptor (EGFR), EGF activates multiple signaling pathways, regulating cell proliferation and differentiation of epithelial cells. Its expression is upregulated during liver regeneration and its overexpression in esophageal (4,5) and gastric cancer (6,7) has been shown to be involved in tumor invasion and poor prognosis. The EGF gene contains an atypical TATA box, polypurine-rich motifs and consensus binding sequences for a number of transcription factors, including nuclear factor-kappa B (NF-kB), gastrin (GAS), activator protein (AP)-1, specificity protein (Sp)-1 and CCAAT/enhancer binding protein (C/EBP) (8). Common genetic variants in the EGF functional region [promoter, exons and 3′ untranslated region (UTR)] may contribute to the inter-individual differences of EGF expression and subsequently disease susceptibility (9). Previous studies have reported that the EGF +61 (A/G) single-nucleotide polymorphism (SNP) in the 5′UTR of the EGF gene (SNP rs444903) is associated with various carcinomas, including gastric cancer (10–13). However, other research did not support the positive results on gastric cancer (14). Since the single locus may not represent the functional region of the gene, it is biologically possible that other functional variants may also be involved in gastric cancer susceptibility. We selected six functional SNPs of the EGF gene besides +61 A/G (rs3756261, rs11568835 and rs4444903 in the promoter region; rs11568943, rs2237051 and rs11569017 in the non-synonymous exon region and rs3733625 in the 3′-UTR region), with a minor allele frequency >0.05 in a Chinese population. To evaluate the effects of these EGF functional SNPs in gastric cancer susceptibility, we conducted genotyping analyses for these SNPs in 387 gastric cancer cases and 392 cancer-free controls in a Chinese population.

## Materials and methods

### Study subjects

This study was approved by the institutional review board of Jiangsu Province Hospital of Chinese Medicine. All subjects were genetically unrelated Han Chinese. A total of 387 gastric cancer patients and 392 cancer-free healthy controls were consecutively recruited from January 2008 to July 2010 in Nanjing city, Jiangsu province, eastern China. The criteria for the recruitment of gastric cancer cases included: a) self-reported Han Chinese; b) local residents for at least 5 years; c) newly histopathologically diagnosed with primary gastric cancer; d) without a previous malignant tumor in any other organ and e) without any anti-tumor therapy prior to recruitment, including chemotherapy and radiotherapy. Any patients who met the above criteria and consented to participate in the study were included. As a result, a total of 400 gastric cancer cases were included in this study with a response rate of 96.8%. The 400 cancer-free controls were collected from hospital visitors who came to the health examination clinic for an annual check-up at the same hospital during the same period. According to the criterion of Lauren’s classification, gastric cancer cases were divided into two subgroups, intestinal type and diffuse type and those with mixed type or not available for classification were denoted with unclassified. A standard questionnaire was administered by trained interviewers to obtain information on demographic data and related risk factors, including tobacco smoking and alcohol consumption. After signing an informed consent form, a 3–5 ml venous blood sample was collected from each subject. A total of 387 gastric cancer cases and 392 unrelated healthy controls were included in this study. Individuals who smoked at least one cigarette per day for over 1 year were considered as smokers and those that had three or more alcohol drinks a week for over 6 months were defined as alcohol drinkers (15–16).

### DNA extraction and genotyping

Venous blood (3–5 ml) was collected from each subject and AxyPrep-96 kit (Axygen, CA, USA) was applied to extract genomic DNA. Polymerase chain reaction-ligation detection reaction (PCR-LDR) methods were used to perform the genotyping. The PCR primers for the seven loci (rs3756261, rs11568835, rs4444903, rs11568943, rs2237051, rs11569017 and rs3733625) were 5′-GCAGATGCTATGGCTGATGA-3′ (forward), 5′-GAAGTGTGATCTGCCCACCT-3′ (reverse) for rs3756261; 5′-GCAAACCTTTTTCCAACCAA-3′ (forward), 5′-AACCTGCACTGACTCTTCGAG-3′ (reverse) for rs11568835; 5′-CATTTGCAAACAGAGGCTCA-3′ (forward), 5′-TGCTCTGGCTGACTTCACTG-3′ (reverse) for rs4444903; 5′-TCCACGCAATGTGTCTGAAT-3′ (forward), 5′-TCAATTTTTAGATGTCACTGAGCAA-3′ (reverse) for rs11568943; 5′-TTCAAGCCTTGTCCTTTCGT-3′ (forward), 5′-GTTTTGCCAATGGATGAACC-3′ (reverse) for rs2237051; 5′-TGCAAAAAGAGGCTTGGAAC-3′ (forward), 5′-TTCAAAATCAGCAAAAGCATAAA-3′ (reverse) for rs11569017; 5′-CACGCCAATGAGGAGTTAAA-3′ (forward), 5′-CAAATTGGGACAACAGTGCTT-3′ (reverse) for rs3733625. PCR was conducted on the ABI 9600 (Applied Biosystems, Foster City, CA, USA) in a system with a total volume of 15 *μ*l, containing 1 *μ*l genomic DNA, 1.5 *μ*l 10X PCR buffer, 0.13 *μ*M each primer, 0.2 mM deoxyribonucleo-tide triphosphate (dNTP), 0.25 *μ*l Taq DNA polymerase (Qiagen GmbH, Hilden, Germany) and 7.5 *μ*l H_2_O. The cycling parameters were: 94°C for 1 min; 35 cycles at 94°C for 10 sec, 56°C for 20 sec, 72°C for 40 sec and a final extension step at 72°C for 3 min. Probe sequences are shown in Table I. The probes for LDR have common phosphorylated 5′-end and 6-carboxyfluorescein (FAM) labeled 3′-end. For each PCR product, the ligation reaction was performed in a final volume of 10 *μ*l, including 2 *μ*l PCR product, 1 *μ*l 10X Taq DNA ligase buffer, 0.02 *μ*M probe mixture, 5 units Taq DNA ligase (New England Biolabs, Beverly, MA, USA) and 6 *μ*l H_2_O. The LDR parameters were as follows: 25 cycles at 94°C for 30 sec and 55°C for 4 min. The LDR reaction products were analyzed on ABI 377 DNA sequencer (Applied Biosystems). To confirm the accuracy of the PCR-LDR genotyping method, 5% samples were randomly selected for direct DNA sequencing for confirmation. PCR-LDR genotyping showed 100% concordance to direct DNA sequencing of the randomly selected PCR products.

### Statistical analyses

The Hardy-Weinberg equilibrium was tested by a goodness-of-fit Chi-square test to compare the observed genotype frequencies with expected ones in the control group. Differences in the distribution of demographic characteristics, selected variables and genotypes of the EGF variants between the gastric cancer cases and controls were evaluated using the two-sided Chi-square test. Logistic regression analysis was employed to estimate crude and adjusted odds ratios (ORs) and 95% confidence intervals (CIs) for the association between EGF genotypes and risk of gastric cancer. Analyses were performed using the software SPSS 16.0 (SPSS Inc., Chicago, IL, USA).

## Results

### Characteristics of study subjects

Gastric cancer cases (387 subjects) and cancer-free controls (392 subjects) were enrolled in our study. The age range for the participants included in the study was 19 to 90 years and the mean ages for the patients and controls were 59.37±13.22 and 50.63±11.74 years, respectively. The percentages of male and female participants were 70.0% and 30.0% in the gastric cancer group and 60.5% and 39.5% in the control group, respectively. The percentages of smokers in the gastric cancer group and the healthy control group were 47.3% and 32.4%, respectively, while the percentages of drinkers were 29.2% and 17.3%, respectively. Of the 387 gastric cancer cases included, 353 (91.2%) patients’ pathological tissues were available for histological type dividing (58 diffuse gastric cancer cases, 280 intestinal gastric cancer cases and 15 gastric cancer cases with mixed histological type) according to the Lauren’s classification. Meanwhile, 34 (8.8%) patients’ pathological tissues were not available for classification (Table II).

### Genotyping distribution and risk of gastric cancer

The seven polymorphisms of EGF genotype distribution in the gastric cancer cases and controls are shown in Table III. The observed genotype distributions for all polymorphisms were in Hardy-Weinberg equilibrium in the controls (P=0.879 for rs3756261, P=0.789 for rs11568835, P=0.099 for rs4444903, P=0.711 for rs11568943, P=0.615 for rs2237051, P=0.570 for rs11569017 and P=0.788 for rs3733625). rs2237051 (Ile to Met) in the exon region demonstrated a significant difference in the genotype distribution between gastric cancer cases and controls. Multiple logistic regression analysis revealed that individuals carrying the AG/GG genotype of rs2237051 (Ile to Met) in the exon region may have an increased risk of gastric cancer (adjusted OR=1.577; 95% CI=1.163–2.138), compared with the wild homozygous AA genotype. The heterozygous AG genotype of rs3733625 in the 3′UTR demonstrated a protective effect (adjusted OR=0.714; 95% CI=0.512–0.996), compared with homozygous AA genotype. However, we did not find an association between the +61 A/G (rs4444903) and gastric cancer. In the stratified analyses, we found that the risk effect of rs2237051 and the protective effect of rs3733625 was evident in subjects older than 50 year old, who were male and drinkers (Table IV). We further evaluated the genetic effects on gastric cancer according to the confirmed histological type (intestinal and diffuse type) and found that the risk effect of the AG/GG genotype of rs2237051 and the protective effect of the AG/GG genotype of rs3733625 were more obvious in intestinal gastric cancer (adjusted OR=1.48, 95% CI=1.08–2.01; adjusted OR=0.70, 95% CI=0.50–0.98, respectively; P<0.05) but not in diffuse gastric cancer (P>0.05; data not shown).

## Discussion

In this case-control study, we investigated the seven functional polymorphisms of the EGF gene with the association of gastric cancer. We found that the variant genotype of EGF rs2237051 AG/GG is associated with an increased risk of intestinal gastric cancer and the variant genotype of EGF rs3733625 AG/GG is associated with a decreased risk of intestinal gastric cancer, compared with their wild-type homozygotes. In the stratified analyses, the effects of the two SNPs were more evident in those older than 50-years old who were male and drinkers.

The EGFR and the EGF-family of peptide growth factors play an important role in the development and progression of diverse carcinoma types, including gastric cancer (10,13,17). Shahbazi *et al*(17) identified an SNP involving A to G transition at position 61 in the 5′UTR of the EGF gene (rs4444903) and the presence of the variant 61A allele led to decreased EGF production in peripheral blood mononuclear cells and decreased susceptibility of malignant melanoma. However, these results were not supported by later studies (18,19). Recently, a number of studies have reported that the SNP rs4444903 is associated with various carcinomas, including gastric cancer (10–13). In the present study, we did not find an association between the rs4444903 variants and the risk of gastric cancer, which is consistent with the results reported by Goto *et al*(14). In addition, we found that SNP rs2237051 (Ile to Met) in the exon region of EGF is associated with an increased risk of gastric cancer (particularly for the intestinal type) and another SNP rs3733625 in the 3′UTR is associated with a decreased risk of gastric cancer (particularly for the intestinal type). However, these results are initial and primitive. Amino acid alteration may influence the spatial conformation of the EGF protein and therefore alter the function of the gene. This may explain the correlation between the SNP rs2237051 of the EGF gene and risk of gastric cancer. Genetic variants in the 3′UTR may influence the stability of mRNA and therfore, the function of a gene. Notably, the two SNPs appeared to confer substantially greater effect in subsets of patients, namely males, elderly individuals and drinkers. Males and elderly individuals are associated with higher cumulative drinking exposure, thus drinking may result in the activation of carcinogenic pathways by the role of the two SNPs of the EGF gene; however, such a conclusion is premature. The two SNPs demonstrated an association with intestinal gastric cancer; however, not for the diffuse type, which may be due to the limited number of confirmed diffuse type gastric cancers. A larger sample size of diffuse type gastric cancer is required to validate this result.

Several limitations in our study need to be addressed: a) the function of the two polymorphisms of the EGF gene in the Chinese population have not been investigated; b) the sample size may limit the statistical power of our study, particularly for subgroup analysis of gastric cancer cases and stratified analysis. We adjusted possible confounding factors, including age, gender, smoking and drinking status, during the process of analysis; c) EGFR gene polymorphisms affecting EGFR activity were not examined.

In conclusion, our study shows that the two polymorphisms (rs2237051 and rs3733625) in the EGF gene may play a role in gastric cancer susceptibility and that drinking status may be a modulating factor. To explore the exact biological mechanism of the two SNPs, further functional studies and larger, well-designed studies, with ethnically diverse populations are required to confirm our findings.

## Figures and Tables

**Table I.** Probe sequences of the studied functional single nucleotide polymorphisms of EGF gene.

SNPs	Probe	Probe sequence (5′-3′)	LDR length
rs3756261	For LDR	P-AAATCCTTCCCAGTTGGGTAGCTCTTTTTTTTTTTTTTTTTTTTTTTTTTTTTTTTTTT-FAM	
	A specific	TTTTTTTTTTTTTTTTTTTTTTTTTTTTTTTTTTATATAAACTTATTAAATATCTCTGT	118 bp
	G specific	TTTTTTTTTTTTTTTTTTTTTTTTTTTTTTTTTTTTATATAAACTTATTAAATATCTCTGC	120 bp
rs11568835	For LDR	P-CAGATGACAGCAATGGAAGGGGCTTTTTTTTTTTTTTTT-FAM	
	G specific	TTTTTTTTTTTTTTTTTCAGCACAATACATTGCCAGCATCC	80 bp
	A specific	TTTTTTTTTTTTTTTCAGCACAATACATTGCCAGCATCT	78 bp
rs4444903	For LDR	P-ACAACCCTTGGATTGGGGCTGAAAGTTTTTTTTTTTTTTTT-FAM	
	G specific	TTTTTTTTTTTTTTTTTTGAAGAACTGATGGAAAGTTCCAGCC	84 bp
	A specific	TTTTTTTTTTTTTTTTGAAGAACTGATGGAAAGTTCCAGCT	82 bp
rs11568943	For LDR	P-TCTCAAGCACTGAGCCTTCAGGACATTTTTTTTTTTTTTTTTT-FAM	
	G specific	TTTTTTTTTTTTTTTTTTTTTCACCGCTACATGTTTTCCCATCTC	88 bp
	A specific	TTTTTTTTTTTTTTTTTTTCACCGCTACATGTTTTCCCATCTT	86 bp
rs2237051	For LDR	P-ATTACTGATGGCATAGCCCAATCTGTTTTTTTTTTTTTTTTTTTTTTTTTTTTTTTT-FAM	
	A specific	TTTTTTTTTTTTTTTTTTTTTTTTTTTTTTTTTTTGCCAGTCCTCTTGTTTACTCTT	114 bp
	G specific	TTTTTTTTTTTTTTTTTTTTTTTTTTTTTTTTTTTTTGCCAGTCCTCTTGTTTACTCTC	116 bp
rs11569017	For LDR	P-CCAGAGCCAGACACGTTTTCCCATCTTTTTTTTTTTTTTTTTT-FAM	
	A specific	TTTTTTTTTTTTTTTTTTTTACCTGCCAACAGCTGATGACCAT	86 bp
	T specific	TTTTTTTTTTTTTTTTTTTTTTACCTGCCAACAGCTGATGACCAA	88 bp
rs3733625	For LDR	P-CTGGACAGAACATTTATATCAGTTTTTTTTTTTTTTTTTTTTTTTTTTTTT-FAM	
	A specific	TTTTTTTTTTTTTTTTTTTTTTTTTTTCAGTTACAAAGTAATTTCTTTGAT	102 bp
	G specific	TTTTTTTTTTTTTTTTTTTTTTTTTTTTTCAGTTACAAAGTAATTTCTTTGAC	104 bp

EGF, epidermal growth factor; SNP, single-nucleotide polymorphism; LDR, ligation detection reaction.

**Table II.** Distribution of selected variables in gastric cancer cases and controls.

Variable	GC cases	Controls	P-value
Number	387	392	
Age (years)	59.37±13.22	50.63±11.74	0.001^a^
Gender			0.005^b^
Male	271 (70.0%)	237 (60.5%)	
Female	116 (30.0%)	155 (39.5%)	
Smoker			0.001^b^
No	204 (52.7%)	265 (67.6%)	
Yes	183 (47.3%)	127 (32.4%)	
Drinker			0.001^b^
No	274 (70.8%)	324 (82.7%)	
Yes	113 (29.2%)	68 (17.3%)	
Histological types			
Intestinal type	280 (72.3%)		
Diffuse type	58 (15.0%)		
Unclassified^c^	49 (12.7%)		

a Non-parametric test.

b Two-sided test.

c Including 15 mixed histological types and 34 unknown. GC, gastric cancer.

**Table III.** EGF gene polymorphisms on the risk of gastric cancer.

Genotype	Gastric cancer cases	Controls	Crude OR (95% CI)	Adjusted OR (95% CI)^a^
Number	387	392		
rs 3756261	383	389		
AA	236	222	1.00	1.00
AG	128	143	0.842 (0.623–1.137)	0.763 (0.552–1.054)
GG	19	24	0.745 (0.397–1.397)	0.629 (0.323–1.225)
AG/GG	147	167	0.828 (0.621–1.104)	0.742 (0.545–1.012)
rs 11568835	366	368		
GG	269	269	1.00	1.00
GA	89	92	0.967 (0.691–1.355)	1.014 (0.708–1.452)
AA	8	7	1.143 (0.409–3.196)	1.090 (0.362–3.275)
GA/AA	97	99	0.980 (0.706–1.359)	1.019 (0.719–1.445)
rs4444903	375	383		
GG	177	204	1.00	1.00
GA	166	142	1.347 (0.997–1.821)	1.325 (0.960–1.829)
AA	32	37	0.997 (0.596–1.667)	1.090 (0.630–1.883)
GA/AA	198	179	1.275 (0.958–1.696)	1.278 (0.942–1.734)
rs 11568943	384	390		
GG	227	220	1.00	1.00
GA	137	144	0.922 (0.684–1.243)	0.890 (0.647–1.225)
AA	20	26	0.746 (0.404–1.374)	0.783 (0.408–1.502)
GA/AA	157	170	0.895 (0.673–1.191)	0.874 (0.644–1.186)
rs2237051	384	387		
AA	162	206	1.00	1.00
AG	193	150	1.636 (1.216–2.201)	1.656 (1.205–2.274)
GG	29	31	1.190 (0.689–2.055)	1.202 (0.672–2.150)
AG/GG	222	181	1.560 (1.174–2.073)	1.577 (1.163–2.138)
rs11569017	365	381		
AA	225	222	1.00	1.00
AT	123	135	0.899 (0.662–1.222)	0.798 (0.574–1.109)
TT	17	24	0.699 (0.365–1.337)	0.683 (0.343–1.363)
AT/TT	140	159	0.869 (0.648–1.165)	0.781 (0.571–1.070)
rs3733625	384	388		
AA	267	243	1.00	1.00
AG	106	127	0.760 (0.557–1.037)	0.714 (0.512–0.996)
GG	11	18	0.556 (0.258–1.201)	0.518 (0.227–1.182)
AG/GG	117	145	0.734 (0.544–0.991)	0.690 (0.501–0.951)

a Adjusted by age, gender, smoking and drinking status. EGF, epidermal growth factor; OR, odds ratio; CI, confidence interval.

**Table IV.** Stratified analyses between rs2237051 and rs3733625 polymorphism and gastric cancer risk.

	rs2237051						rs3733625					
Variable	GC cases		Controls		^a^Adjusted OR (95%CI)		GC cases		Controls		^a^Adjusted OR (95%CI)	
					
	AA	AG/GG	AA	AG/GG	AA	AG/GG	AA	AG/GG	AA	AG/GG	AA	AG/GG
Age (years)												
≤50	32	45	106	91	1.00	1.64 (0.96–2.79)	51	26	129	71	1.00	0.93 (0.53–1.61)
>50	126	172	100	89	1.00	1.53 (1.06–2.21)	211	87	113	74	1.00	0.63 (0.43–0.93)
Gender												
Male	107	161	126	108	1.00	1.76 (1.23–2.50)	190	78	144	92	1.00	0.64 (0.44–0.93)
Female	55	61	80	73	1.00	1.22 (0.75–1.97)	77	39	99	53	1.00	0.95 (0.57–1.58)
Smoker												
No	96	107	136	126	1.00	1.20 (0.83–1.74)	139	64	164	98	1.00	0.77 (0.52–1.14)
Yes	66	115	70	55	1.00	2.22 (1.39–3.53)	128	53	79	47	1.00	0.70 (0.43–1.13)
Drinker												
No	117	154	163	156	1.00	1.38 (0.99–1.90)	189	82	207	114	1.00	0.79 (0.56–1.11)
Yes	45	68	43	25	1.00	2.60 (1.40–4.83)	78	35	36	31	1.00	0.52 (0.28–0.97)

a Adjusted by age, gender, smoking and drinking status. GC, gastric cancer; OR, odds ratio; CI, confidence interval.
